# Inclusive orchestral music therapy according to the Euterpe Method: a multimodal framework for neurodevelopmental disorders

**DOI:** 10.3389/fneur.2025.1612955

**Published:** 2025-10-02

**Authors:** Tommaso Liuzzi, Fiammetta D’Arienzo, Susanna Staccioli, Rita Faraj Slaïby, Maroun Bou Sleiman Harb, Miled Tarabay, Roberto Giuliani, Teresa Chirico, Donatella Lettori, Enrico Castelli

**Affiliations:** ^1^Unit of Neurorehabilitation, Bambino Gesù Children’s Hospital, IRCCS, Rome, Italy; ^2^Santa Cecilia Conservatory of Music, Rome, Italy; ^3^Euterpe APS Cultural Association, Rome, Italy; ^4^Insieme Association, Sahel Alma, Lebanon; ^5^School of Music and Performing Arts, Holy Spirit University of Kaslik, Jounieh, Lebanon; ^6^Faculty of Law, Université La Sagesse, Furn El Chebbak, Lebanon; ^7^Neurorehabilitation Research Area, Bambino Gesù Children’s Hospital, IRCCS, Rome, Italy

**Keywords:** inclusive orchestral music therapy, Euterpe Method, auditory-motor, neurodevelopmental disorders, cerebral palsy, autism spectrum disorder, inclusion

## Abstract

Neurodevelopmental disorders (NDD), as defined by DSM-5-TR and CDDR, comprise heterogeneous early-onset conditions involving executive dysfunction, motor planning deficits, language impairments, and socio-emotional dysregulation. Evidence from neuroimaging and clinical studies suggests that music-based interventions may engage distributed neural networks—including fronto-striatal, temporo-parietal, limbic, and brainstem circuits—through predictive timing, cross-modal synchronization, and adaptive plasticity. However, clinical translation has been hindered by methodological heterogeneity, insufficient standardization, and reduced reproducibility, together with limited integration of clinical, functional, and neurophysiological indicators, absence of unified protocols combining individualized and orchestral modules with explicit transfer mechanisms, and insufficient monitoring of fidelity and multisite feasibility. This perspective proposes the IncluSive Orchestral mUsic therapy accordiNg to the euterpe methoD (I-SOUND), a clinically adapted orchestral framework structured to integrate three complementary modules: Individual Music Therapy (IMT), an Orchestral Music Therapy module (OMT), and a Multidirectional and Iterative Transfer Process (MIT-P). Developed from the progressive refinement of the Euterpe Method and the pediatric EM Active algorithm, the model is intended to target specific neurofunctional domains and to explore generalization to everyday contexts. A two-phase evaluation—comprising an observational study followed by a randomized controlled trial—is planned to assess feasibility, fidelity, sustainability, and clinical applicability in heterogeneous NDD populations. Particular attention is given to the methodological challenge of balancing ethical inclusion with internal validity. No efficacy claims are advanced, as the framework requires empirical verification before clinical conclusions can be drawn.

## 1 Introduction

Neurodevelopmental disorders (NDD), as defined in the Diagnostic and Statistical Manual of Mental Disorders, Fifth Edition, Text Revision (DSM-5-TR) (1) and the Clinical Descriptions and Diagnostic Requirements for ICD-11 Mental, Behavioral and Neurodevelopmental Disorders (CDDR) (2), are early-onset, heterogeneous conditions affecting executive function, motor planning, language, and socio-emotional regulation. Prevalence estimates vary owing to differences in diagnostic criteria, assessment methods, and systemic disparities, underscoring the need for rigorous, inclusive research designs (3).

From a neurofunctional perspective, the orchestra can be framed as a multisensory relational system characterized by temporal synchronization, hierarchical coordination, and functional differentiation. When clinically contextualized, it may engage motor, cognitive, and socio-affective systems in line with adaptive plasticity (4). Neuroimaging and neurophysiology indicate that individual and ensemble practice recruit distributed cortical–subcortical networks mediating auditory, motor, and affective integration (5–8), consistent with predictive timing, sensorimotor coupling, interpersonal synchronization, and neuromodulatory processes. Because most findings derive from neurotypical samples, these mechanisms remain hypotheses requiring targeted verification in NDD.

Clinically adapted orchestral music-making may operate as a multimodal enriched therapeutic environment supporting combined sensory, motor, and social stimulation (9, 10). However, persistent gaps include protocol heterogeneity, limited standardization and reproducibility, and insufficient integration of clinical, functional, and neurophysiological indicators. Designs must also balance ethical inclusion with internal validity where baseline variability and comorbidities complicate interpretation. Current literature lacks unified protocols combining individual and orchestral music therapy with explicit transfer mechanisms, fidelity monitoring, and multisite feasibility assessments (11–13).

The present framework results from progressive refinement of the Euterpe Method in pediatric and adolescent NDD cohorts, where diagnostic criteria, outcome indicators, and modular structures were operationalized. Prior studies reported adaptability in home-based telerehabilitation (14), targeted interventions for cerebral palsy (CP) (15), and methodological structuring of neurofunctional algorithms (16), leading to the EM Active procedural model and related algorithms for diverse contexts. Building on this platform, individual and orchestral modules were clinically expanded for adolescents and young adults with NDD. The IncluSive Orchestral mUsic therapy accordiNg to the euterpe methoD (I-SOUND) model is thus proposed as a methodological framework intended to: reconcile inclusion with methodological integrity, integrate multimodal neurofunctional targets, and explore reproducibility in heterogeneous clinical populations.

I-SOUND comprises: (i) Individual Music Therapy (IMT), targeting domain-specific outcomes; (ii) Orchestral Music Therapy (OMT), an ensemble-based module promoting interpersonal synchronization and hierarchical coordination; and (iii) the Multidirectional and Iterative Transfer Process (MIT-P), a regulatory mechanism for cross-context generalization and consolidation. The model is evaluated through a two-phase design: Phase 1, a longitudinal observational protocol adhering to Strengthening the Reporting of Observational Studies in Epidemiology (STROBE) (17); and Phase 2, a randomized controlled trial (RCT) aligned with the Consolidated Standards of Reporting Trials (CONSORT) (18, 19); interventions are described using the Template for Intervention Description and Replication (TIDieR) (20).

## 2 State of the art in music-based interventions for neurodevelopmental disorders

### 2.1 Neurofunctional rationale

Ensemble music-making constitutes a temporally structured, multimodal environment with differentiated roles and hierarchical coordination (9, 10). Computational and theoretical models suggest engagement of predictive coding for social synchronization and sensorimotor coupling (8, 21). In typically developing populations, activity spans fronto-striatal, temporo-parietal, limbic, and brainstem circuits contributing to entrainment and alignment (5–7, 22).

Structured music training has been associated with experience-dependent plasticity and enriched-environment effects across motor, cognitive, and socio-affective systems (4, 10, 12, 23, 24). Reported neurostructural adaptations include enhanced interhemispheric connectivity, reorganization of motor regions, and strengthened audio–motor coupling in pediatric and adult cohorts (7, 25, 26). These processes are salient during the 9–25-year developmental window, when sensorimotor, executive, and socio-affective systems show heightened susceptibility to experience-driven modulation (27–29).

Overall, these findings support the hypothesis that clinically adapted orchestral practice could function as a multimodal enriched environment during sensitive developmental windows. Within this perspective, I-SOUND is introduced as an exploratory framework to examine such hypotheses in NDD through sequential individual–ensemble modules and regulatory transfer mechanisms aimed at ecological validity and reproducibility (15, 16).

### 2.2 Neurobiological substrates of musical interaction

Evidence on neural substrates of musical interaction in NDD is limited; most data derive from neurotypical or mixed samples. Neuroimaging and electrophysiology suggest that ensemble music can enhance audio–motor coupling, engage mirror neuron systems, and modulate dopaminergic and serotonergic pathways, although current evidence derives predominantly from cross-sectional studies and remains preliminary (4–7). Predictive coding accounts posit minimization of prediction errors in melody, rhythm, and harmony, potentially improving synchrony (8). Groove-rich music and salient visual cues may promote motor engagement and alignment (21), recruiting fronto-striatal circuits, insula, and anterior cingulate cortex—regions implicated in timing, emotion, and social regulation. Group-based musical practices have also been associated with changes in neurotrophic and stress-related indices, but these findings remain exploratory and are not the focus of the present program, which prioritizes clinically validated functional outcomes. These mechanisms remain plausible targets requiring structured testing in NDD, which I-SOUND is designed to explore.

### 2.3 Ensemble-based approaches without controlled clinical validation

Several ensemble programs were conceived primarily for psychosocial inclusion rather than as trial-ready clinical protocols; their evidence base is largely observational or pilot-level, including El Sistema (22, 30–33), Nordoff-Robbins (34), Strokestra (35, 36), Esagramma (37), AllegroModerato (38), and community-oriented frameworks such as Community Music Therapy (39–45). These initiatives prioritize expressive and relational aims and provide useful observations—for example, groove-rich repertoires and salient visual interaction may support coordination and prosocial behaviors (21). Yet most lack standardized eligibility criteria, fidelity thresholds, prespecified endpoints, or multisite procedures with blinded assessment in pediatric or transitional-age NDD cohorts (46, 47).

In contrast, I-SOUND may be described as a clinical framework that proposes to translate these inclusive premises into a trial-ready structure, organized around the modular sequence IMT-OMT-MIT-P. Its design is intended to align with DSM-5-TR/CDDR diagnostic criteria, to incorporate predefined fidelity metrics with an intraclass correlation coefficient (ICC) ≥ 0.80, and to follow a two-phase methodology (Phase 1 STROBE; Phase 2 CONSORT) aimed at enhancing transparency, reproducibility, and clinical applicability. In this context, I-SOUND is introduced as a tentative clinical framework differing by: (i) a standardized IMT–OMT sequence regulated by MIT-P; (ii) TIDieR-compliant specification with fidelity thresholds (ICC ≥ 0.80) and replication materials; (iii) a two-phase design (Phase 1 STROBE; Phase 2 CONSORT) with stratified randomization and blinded outcomes; and (iv) multicenter feasibility through predefined adaptations and accredited provider training (20, 46, 47).

### 2.4 Evidence in neurodevelopmental disorders

Music-based interventions (MBI) in NDD have reported effects in motor, language, and socio-emotional domains, particularly in autism spectrum disorder (ASD), CP, and selected genetic syndromes (46, 48–50). Modalities include Rhythmic Auditory Stimulation (RAS), therapeutic singing, and instrumental training, with substantial heterogeneity in dosage, duration, and complexity. Reviews indicate that music may support functional and structural adaptations, and that ensemble formats could contribute to socially mediated plasticity (10, 23). Nonetheless, most investigations are modality-specific and seldom integrate individualized and collective modules within stratified cohorts. No controlled studies have validated an orchestral framework explicitly combining IMT, OMT, and MIT-P in NDD, nor examined multisite feasibility with blinded assessment and fidelity monitoring.

### 2.5 Gap analysis

Despite growing interest, the MBI literature in NDD is marked by methodological variability, short follow-up, and limited standardization (46, 51, 52). Incorporating predictive-processing models and groove-mediated engagement within structured designs may clarify ensemble-driven plasticity. Yet interventions rarely include predefined fidelity metrics, stratification strategies, or blinded assessments. To our knowledge, no published protocol integrates IMT, OMT, and MIT-P within a unified, developmentally calibrated orchestral framework with systematic clinical monitoring and multicenter evaluation. I-SOUND was therefore developed as a cautious methodological proposal aligned with DSM-5-TR/CDDR, operationalizing neurofunctional premises through MIT-P cycles and sequencing modules to accommodate developmental transitions. Generalization is conceptualized as near and far transfer, operationalized through time-defined MIT-P cycles to support consolidation and cross-context application.

## 3 Methods and study design

This two-phase program investigates I-SOUND in individuals with NDD (DSM-5-TR, CDDR) (1, 2), aged 9–25 years in Phase 1 and 18–25 years in Phase 2, eligible if functionally able to join structured sessions. Exclusion criteria include uncorrected sensory deficits, unstable conditions, contraindications to group participation, and profiles incompatible with standardized testing as detailed in Table 1. The developmental window was chosen for sensitivity to experience-dependent plasticity across motor, cognitive, and socio-affective systems (27–29).

**Table 1:** I-SOUND study plan aligned with STROBE, CONSORT, and TIDieR.

Domain	Phase 1	Phase 2
Study design	20-week longitudinal observational study modeling therapeutic processes and refining parameters under ecological conditions.	RCT with five timepoints (T0: baseline; T1: week 10; T2: week 20; T3: 12-month follow-up; T4: 24-month follow-up). Variable block randomization; stratification by diagnosis and functional level (GMFCS, VABS-3 Motor Skills (55, 56)); allocation concealment with opaque sealed envelopes; blinded assessors and analysts.
Operational setting	Extra-hospital facilities with verified acoustic suitability and safety, supporting structured multimodal interventions for NDD and ensuring continuity of care through the I-SOUND model. Sessions are delivered in hospital-based rehabilitation units, conservatory auditoria, and community-based centers. All settings are accessible and acoustically treated according to safety standards (RT60 < 1.0 s; LAeq ≤ 85 dB).	
	Conducted at a social promotion association in the northern periphery of Rome, Italy.	Conducted at a Conservatory of Music in Rome, Italy, with dedicated spaces that meet acoustic and logistical requirements for orchestral sessions and independent assessment rooms; site procedures harmonized for multi-site reproducibility.
Multidisciplinary team	Certified conductor–music therapist with responsibility for orchestral direction and therapeutic integration; neurologist, neuropsychiatrist, psychologist, and researchers; professional musicians and music therapists trained in the Euterpe Method; trained volunteer musicians in supportive performance roles only, under continuous supervision and without autonomous clinical responsibilities. Neurotypical peers may be included for inclusive joint practice, contingent on training and protocol adherence.	
Participants & eligibility	Inclusion: 9–25y, NDD (DSM-5-TR, CDDR) (1, 2); ability to participate in structured sessions. Exclusion: uncorrected severe sensory deficits, unstable conditions, contraindications to group participation.	Inclusion: 18–25y, NDD; standardized profiling for eligibility. Exclusion: profiles incompatible with standardized testing protocols.
Intervention modules	IMT: predictive timing, multimodal sensorimotor integration, sustained attention. OMT: ensemble role differentiation, temporal synchronization, multimodal cueing. MIT-P: multidirectional transfer of competences. Each module: delivered once per week over 20 weeks (IMT: 20 × 60 min; OMT: 20 × 90 min), with sessions scheduled 1–5 days apart.	Same modular structure with calibrated frequency, intensity, and role assignment; standardized orchestral adaptations for multi-site reproducibility, with assessments scheduled at T0, T1, and T2.
Adaptive strategies	Codified adjustments: instrumental modifications (supports, ergonomic devices), reduced visual density in PTC scores, role rotation, structured group dynamics. Detailed in TIDieR (Supplementary Table 1).	Identical adaptations with fidelity monitoring; corrective actions predefined.
Research questions	Primary: How do functional domains (motor, cognitive, socio-communicative, emotional-regulatory, motivational, synchronization) evolve longitudinally under I-SOUND intervention, as measured through weekly EMA-T and EMA-P? Secondary: What is the cross-observer consistency of evaluations, and which individual and orchestral configuration factors influence feasibility, adherence, and scalability?	Primary: Does I-SOUND improve motor coordination, executive functioning, and emotional regulation compared to baseline? Secondary: Which moderators (diagnosis, baseline level, instrumental role) influence the efficacy and persistence of effects at T4 = follow-up timepoint?
Outcome measures	Primary: EMA-T (therapist-reported module, compiled ≤24 h by a clinical evaluator distinct from the intervention provider, operating in blinded conditions where feasible) and EMA-P (caregiver 24–48 h) (53), weekly ×20 weeks, across motor, cognitive, socio-communicative, emotional-regulatory, motivational, and synchronization domains. Secondary: feasibility and adherence.	Primary: Δ(T2–T0) in age-standardized motor composite: BOT-2 (9–21y) (57, 58) or MABC-3 (≥21y) (59). Secondary: manual dexterity (BBT (60)), executive functions (BRIEF-2/BRIEF-A (61, 62)), adaptive functioning (VABS-3 (55)), quality of life (PedsQL (63)). Exploratory: wearables Xsens DOT, MusicGlove (64, 65), assessing feasibility and sensitivity to change; excluded from efficacy.
Assessors & timing	EMA-T: independent blinded clinician. EMA-P: caregiver. ICC target ≥0.80.	Independent blinded assessors at T0–T4, within 24 h of each timepoint.
Sample size & ethics	N = 22 NDD individuals plus 18 musicians (7 music therapists, 5 professional musicians, 6 trained volunteers), all certified in the Euterpe Method; ecological recruitment without formal power calculation; voluntary participation with caregiver consent; OPBG REC approval; compliant with the Declaration of Helsinki (83) and ICH-GCP.	N = 20 NDD individuals, with approximately 50% integrated with orchestral peers; sample size defined for effect-size estimation with confidence intervals (precision, not hypothesis testing). Registered on ClinicalTrials.gov; compliant with the Declaration of Helsinki (83), ICH-GCP, and OPBG REC approval.
	Transparency notes: This exploratory phase is not powered for hypothesis testing. Precision metrics from effect-size estimates and confidence intervals will inform the sample size for the subsequent multicenter RCT.	
Statistical plan	Descriptive statistics, regression models for EMA trajectories; mixed-effects models for repeated measures.	ITT; multiple imputation (≥20 replicates, MAR/MNAR sensitivity); ANCOVA or MMRM for continuous outcomes; hierarchical Holm–Bonferroni correction; moderator and subgroup analyses (diagnosis, baseline, instrumental role). Sensitivity analyses comparing complete-case vs. imputed datasets.
Data & integrity	Paper-based anonymized data collection; REC-approved storage.	REDCap-based audit trail, pseudonymized identifiers, source data verification, database lock. Transparency ensured by ClinicalTrials.gov registration and TIDieR (20) documentation.
Safety & fidelity	Session adherence ≥80%; ICC ≥ 0.80 on video double-coding; incident log; stop/adjust rules predefined.	Same criteria plus safety board monitoring; corrective protocols implemented.
Provider training	≥60 h in Euterpe Method (IMT, OMT, MIT-P); includes neurofunctional rationale, adaptive procedures for NDD.	Ongoing supervision and inter-rater calibration for blinded assessments.

The table summarizes all primary, secondary, and exploratory outcomes across study phases, in accordance with CONSORT, SPIRIT, and TIDieR guidelines. Instruments were selected to ensure ecological validity in Phase 1 and standardized comparability in Phase 2, while accommodating the heterogeneity of NDD. For each instrument, the table specifies the target population, age range, and assessment timepoints. In cases where normative data are limited (e.g., Vineland Adaptive Behavior Scales, Third Edition *—* Motor Skills domain for participants >9 years 11 months), scores will be used exclusively for descriptive purposes and within-sample stratification, without external normative comparison. The combination of the Vineland Adaptive Behavior Scales, Third Edition *—* Motor Skills domain and the Gross Motor Function Classification System provides complementary perspectives—adaptive-ecological and clinical-functional—on gross motor ability. Wearable devices (Xsens DOT, MusicGlove) are included as exploratory tools to complement standardized assessments with high-frequency biomechanical data; their use is restricted to selected sessions, and they are not considered primary endpoints. This structure ensures full transparency in the operationalization of outcomes, supporting replicability and methodological rigor in the evaluation of the I-SOUND model in NDD populations.

MIT-P, EMA-T/EMA-P are MIT-P monitoring tools in Phase 1; Phase 2 relies exclusively on blinded standardized assessments (see Section 3).

ANCOVA, Analysis of Covariance; BBT, Box and Block Test; BOT-2, Bruininks–Oseretsky Test of Motor Proficiency, Second Edition; BRIEF-2, Behavior Rating Inventory of Executive Function, Second Edition; BRIEF-A, Behavior Rating Inventory of Executive Function – Adult Version; CDDR, Clinical Descriptions and Diagnostic Requirements for ICD-11 Mental, Behavioral and Neurodevelopmental Disorders; CONSORT, Consolidated Standards of Reporting Trials; DSM-5-TR, Diagnostic and Statistical Manual of Mental Disorders, Fifth Edition, Text Revision; EMA-P, Ecologic Momentary Assessment – Parent Version; EMA-T, Ecologic Momentary Assessment – Therapist Version; GMFCS, Gross Motor Function Classification System; Holm–Bonferroni, Holm–Bonferroni method for multiple comparison correction; ICC, Intraclass Correlation Coefficient; ICH-GCP, International Council for Harmonization – Good Clinical Practice; IMT, Individual Music Therapy; ITT, Intention-to-Treat; I-SOUND, IncluSive Orchestral mUsic therapy accordiNg to the euterpe methoD; LAeq, Equivalent continuous sound level; MABC-3, Movement Assessment Battery for Children, Third Edition; MAR, Missing at Random; MIT-P, Multidirectional and Iterative Transfer Process; MMRM, Mixed-Effects Model for Repeated Measures; MNAR, Missing Not at Random; NDD, Neurodevelopmental Disorders; OMT, Orchestral Music Therapy; OPBG, Ospedale Pediatrico Bambino Gesù; PedsQL, Pediatric Quality of Life Inventory Generic Core Scales; PTC, Personalized Therapeutic Compositions; RCT, Randomized Controlled Trial; REC, Research Ethics Committee; REDCap, Research Electronic Data Capture; RT60, Reverberation time (sound decay by 60 dB); STROBE, Strengthening the Reporting of Observational Studies in Epidemiology; TIDieR, Template for Intervention Description and Replication; VABS-3, Vineland Adaptive Behavior Scales, Third Edition.

Phase 1, a 20-week longitudinal observational study aligned with STROBE (17), employs weekly Ecological Momentary Assessments (EMA) (53), developed by clinical experts: therapist-reported (EMA-T), compiled within 24 h by an evaluator distinct from the intervention provider under blinded conditions, and caregiver-reported (EMA-P), completed within 48 h, encompassing motor, cognitive, socio-communicative, emotional-regulatory, motivational, and synchronization domains. An independent co-rating is performed on a 20–25% sample of sessions; fidelity requires an ICC ≥ 0.80. Each weekly cycle comprised one IMT session (60 min) and one OMT session (90 min), scheduled 1–5 days apart, resulting in a total of 20 sessions per module across the 20-week program. Descriptive and regression analyses map trajectories; procedures and fidelity safeguards appear in Supplementary materials 1, 2 and Table 1.

Phase 2 is an RCT aligned with CONSORT (18, 19), with assessments scheduled at T0 (baseline), T1 (week 10), T2 (week 20), T3 (12-month follow-up), and T4 (24-month follow-up). The choice of 10- and 20-week intervals allows detection of short- and medium-term changes, while annual and biennial follow-ups provide information on maintenance and long-term trajectories, consistent with literature on outcome monitoring in NDD (54). Randomization uses variable blocks stratified by diagnosis and functional level through the Gross Motor Function Classification System and the Motor Skills domain of the Vineland Adaptive Behavior Scales, Third Edition (55, 56). Allocation concealment is performed with independent opaque envelopes; assessors and analysts remain blinded. Outcomes include the Bruininks–Oseretsky Test of Motor Proficiency, Second Edition for ages 9–21 years (57, 58) or the Movement Assessment Battery for Children, Third Edition for ages above 21 years (59) as the age-standardized motor composite, which represents the primary endpoint Δ(T2–T0). Secondary outcomes include the Box and Block Test (60), the Behavior Rating Inventory of Executive Function, Second Edition and Behavior Rating Inventory of Executive Function–Adult Version (61, 62), the Vineland Adaptive Behavior Scales, Third Edition (55), and the Pediatric Quality of Life Inventory (63). Exploratory wearables such as Xsens DOT and MusicGlove (64, 65) provide parallel monitoring of feasibility and sensitivity to change but are excluded from efficacy testing. Further implementation details are in Table 1.

Variability and bias are managed through age-calibrated tools, stratification, blinded assessments, and a Research Electronic Data Capture (REDCap) system with role-based access control and complete audit trail. Adaptive strategies including instrumental modifications, role rotation, reduced visual density of Personalized Therapeutic Compositions (PTC) scores, and group management are pre-specified for replicability and codified in Table 1 and the TIDieR’s Supplementary Table 1. Analyses include intention-to-treat, multiple imputation with at least 20 replicates and both missing-at-random and missing-not-at-random sensitivity, analysis of covariance or mixed-effects model for repeated measures, hierarchical Holm–Bonferroni, as well as moderator and subgroup analyses by diagnosis, baseline profile, and instrumental role. Sensitivity analyses will compare complete-case and imputed datasets. Safety monitoring includes predefined stop and adjust rules, adverse-event logs, fidelity thresholds of at least 80% adherence, and inter-rater reliability with ICC ≥ 0.80 on double coded video material. Providers receive at least 60 h of training in the Euterpe Method across IMT, OMT, and MIT-P to ensure intervention fidelity. Transparency is reinforced by registration on ClinicalTrials.gov and TIDieR documentation in Table 1 and the Supplementary Table 1.

### 3.1 Modular structure and clinical adaptation

**Figure 1:**
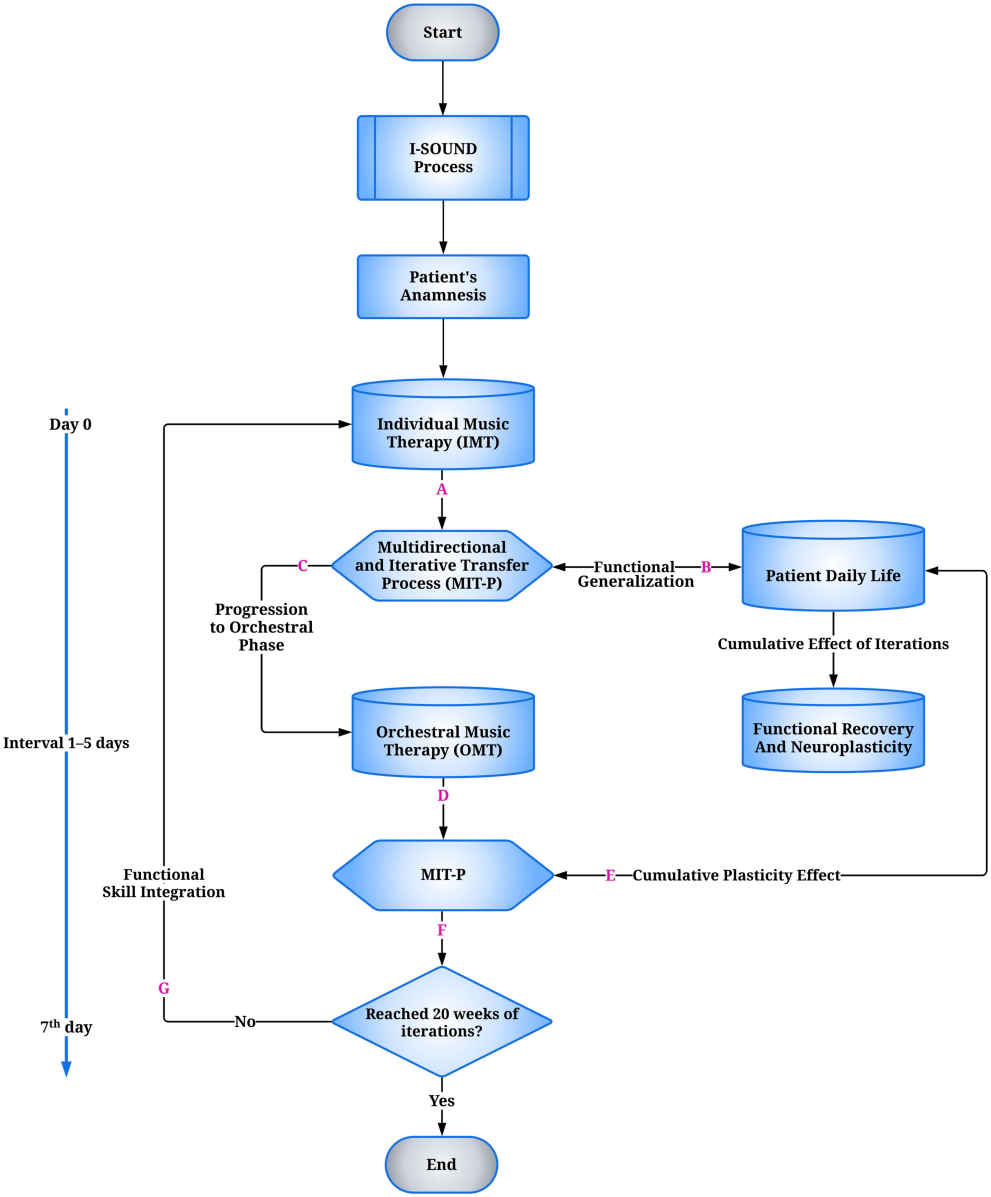
Flowchart of the I-SOUND model for neurodevelopmental disorders (NDD). The process begins with the clinical-functional assessment, which guides the individualized intervention. This is followed by Individual Music Therapy (IMT), targeting predictive timing, multimodal sensorimotor integration, and sustained attention. At the end of each cycle, the Multidirectional and Iterative Transfer Process (MIT-P) (A) regulates functional outcomes: if transferable, they are functionally transposed into ecological daily-life contexts (B); if adequate, they allow progression to the orchestral phase (C). Orchestral Music Therapy (OMT) sessions are not co-delivered on the same day as IMT but are scheduled exclusively after an inter-session latency of 1–5 days, during which MIT-P mediates the stabilization and inter-contextual transfer of functional gains. Within OMT, MIT-P supports intermodular coherence and skill integration (D). Both trajectories converge toward functional recovery and experience-dependent neuroplasticity (E). A decision node (F) verifies the predefined temporal threshold of 20 weeks of iterative cycles: if met, the process concludes (End); if not, the loop restarts (G). The temporal structure includes micro cycles (Day 0, 1–5-day interval) and weekly macrocycles (7th day). IMT, Individual Music Therapy; OMT, Orchestral Music Therapy; MIT-P, Multidirectional and Iterative Transfer Process.

I-SOUND, a clinically intensive extension of the Euterpe Method, is structured according to TIDieR (20) and integrates principles of experience-dependent plasticity (29), stratified functional targeting, and adaptive musical codification (Figure 1). IMT and OMT are derived from the EM Active algorithm, initially designed for pediatric cohorts and later adapted for adolescents and young adults with NDD. EM Active functions as a clinical device supporting adaptive resilience and longitudinal functional targeting, maintaining methodological continuity across developmental phases (15, 16).

#### 3.1.1 Modules and methodological chronology

- IMT: 60-min individual sessions targeting predictive timing, multimodal sensorimotor integration, and sustained attention.
- OMT: 90-min orchestral sessions extending IMT-acquired skills into hierarchical ensemble structures. Clinically adapted conducting techniques—gestural segmentation, temporal modulation, multimodal cueing, and role distribution—are combined with therapeutic materials, including Compositional Sound Interventions (CSI) and PTC, to align orchestral execution with individualized neurofunctional objectives (16). Replicable adaptations include adjustable chin/hand supports, low-density color-coded PTC staves, programmed role rotation, and scheduled ‘quiet breaks’ for attention regulation, all formalized in Table 1 and Supplementary Table 1.
- MIT-P: a transversal regulatory mechanism sustaining inter-modular coherence and consolidation through cycles of active practice, consistent with adaptive motor-learning models and therapeutic context modulation (66). Evidence indicates transfer to untrained tasks even without spatiotemporal similarity (67). MIT-P integrates automatic and reflective components (low/high-road transfer) (68–70), supported by experience-modulated cortical plasticity and transient disinhibition dynamics (24).

## 4 Discussion

The present framework derives from the progressive refinement of the Euterpe Method in pediatric and adolescent NDD cohorts, where clinical criteria, assessment tools, and modular structures were delineated. Its adaptability has been documented in telerehabilitation protocols (14), targeted interventions for CP (15), and the methodological formalization of neurofunctional procedures (16). This formalization evolved into four complementary algorithms—EM Hospital-based, EM Active, EM Receptive, and EM Telerehabilitation—that, despite different contexts and aims, provided a unified platform for clinical translation. On this foundation, orchestral adaptations were developed through clinically oriented conducting techniques and dedicated compositional materials, forming the basis for OMT. Within this modular progression, the I-SOUND model is framed as a structured extension designed to address persistent methodological challenges: balancing ethical inclusion with internal validity (STROBE, CONSORT), integrating individual and orchestral domains through MIT-P mechanisms, and pursuing reproducibility in heterogeneous NDD populations, consistent with TIDieR criteria (17–20).

### 4.1 Theoretical framing and methodological positioning

I-SOUND is a modular, cyclic framework addressing three recurrent gaps: limited integration of individual and ensemble modalities, absence of a formalized transfer mechanism, and lack of standardized, reproducible procedures. Its architecture aligns with neurofunctional substrates implicated in adaptive plasticity, predictive timing, and cross-modal synchronization (71). The design seeks to balance ethical inclusion with internal validity through adaptive eligibility and implementation strategies. Within this framework, MIT-P coordinates transfer between IMT and OMT via temporally defined practice sequences, supporting learning, consolidation, and fidelity across heterogeneous profiles. Distinct from community-oriented programs, I-SOUND incorporates prespecified fidelity thresholds (e.g., ICC ≥ 0.80), stratified randomization, blinded assessment, multicenter planning, and systematic video coding by multiple trained raters to mitigate observational bias (72).

### 4.2 Scientific and clinical implications

IMT targets predictive timing and multimodal sensorimotor integration; OMT extends these capacities in structured ensemble contexts. MIT-P ensures inter-modular coherence through repeated cycles (66), with potential improvements in untrained tasks (67), conceptualized as near and far transfer and integrating automatic and reflective components (68–70), hypothetically engaging cortical plasticity and transient disinhibition (24). Collectively, modules could activate bilateral audio–motor networks, including supplementary motor area, premotor cortex, basal ganglia, and cerebellum, consistent with beat-based mechanisms (73, 74). Hierarchical synchronization and role differentiation, interpreted within predictive-coding models, may reduce error signals and support emotional regulation and interpersonal coordination (8). Observations from collective music-making in neurotypical cohorts point to improvements in motor control, emotion regulation, and plasticity, which require empirical verification in NDD. High-groove music and visual social cues have been associated with increased movement energy and coordination in ensembles (21). Plasticity studies suggest that instrumental practice can modulate fronto-parietal and cerebellar activity, with auditory and striatal measures predicting learning rate; preliminary evidence indicates modulation of neurotrophic and stress-related biomarkers, although findings remain exploratory (10, 23, 75, 76). Caregiver involvement, ecological momentary assessment, and wearable sensors are positioned to increase ecological validity, personalize parameters, and enhance sensitivity to change; wearables remain exploratory and excluded from efficacy analyses.

### 4.3 Methodological strengths and core limitations

The biphasic design—observational modeling followed by RCT—aims to balance ecological validity with controlled hypothesis testing (77, 78). Strengths include validated multidomain assessments (79), fidelity controls, and modular implementation adaptable to diverse contexts (80). Preliminary Italy–Lebanon experience may support transcultural replication, pending empirical confirmation. The inclusion of EMA-T/EMA-P (Supplementary materials 1, 2) and TIDieR (Supplementary Table 1) contributes to reproducibility, sensitivity to change, and transparency, aligning with STROBE, CONSORT, and TIDieR.

Limitations include the absence of harmonized international protocols (81), resource demands (specialized personnel, adapted instruments, flexible spaces) (82), and the methodological challenge of balancing ethical inclusion with internal validity. Another limitation is the lack of accredited training for the conductor–music therapist role, requiring structured pathways. Current mitigation—modular equipment, inter-institutional collaborations, and training initiatives—remains partial.

### 4.4 Operational challenges and safety management

Safety is addressed through pre-session health checks, continuous therapist monitoring, and post-session debriefings, with adaptive procedures allowing modification or suspension in cases of instability (78, 83). Incidents are centrally logged and reviewed weekly by the clinical team. To limit bias, systematic video recording with multi-rater cross-coding improves reliability, although resource-intensive. Operational constraints persist (specialized personnel, adapted instruments, institutional variability) (82); current mitigation via modular setups and shared facilities provides only partial relief. Accredited training and harmonized protocols remain prerequisites for scaling.

### 4.5 Future directions

The modular structure may be adapted to specific NDD subgroups (71, 84). Wearable technologies and longitudinal EMA could enhance ecological monitoring. Given that the current program already includes structured follow-up assessments up to 24 months, subsequent research should investigate whether extending monitoring beyond this timeframe is clinically informative (85). In future applications, if the intervention is requested for periods longer than 20 weeks, additional follow-up assessments will be planned according to treatment course and outcomes observed at earlier timepoints, allowing data-driven adaptation of longitudinal monitoring (86). Furthermore, planned developments include multicenter implementation with harmonized standards, accredited training for the conductor–music therapist role, and a standardized starter-kit (instrument set, compositional/therapeutic library, fidelity manual). International collaborations may facilitate scalability while maintaining cultural adaptability.

## 5 Conclusion

I-SOUND is a theory-based, clinically adaptable framework integrating IMT, OMT, and MIT-P. Its biphasic evaluation—observational modeling followed by RCT—seeks to balance inclusion with internal validity and scalability. Feasibility, sustainability, and applicability will be assessed to develop standardized multimodal strategies for NDD. In essence, I-SOUND is a structured clinical hypothesis requiring validation through controlled studies; the present framework provides a coherent basis for such verification without inferring outcomes.

## Data Availability

The original contributions presented in the study are included in the article/Supplementary material, further inquiries can be directed to the corresponding author.
