# Supplementary material for: Inclusive orchestral music therapy according to the Euterpe Method: a multimodal framework for neurodevelopmental disorders
Source: Front Neurol. 2025 Oct 2;16:1612955. doi: 10.3389/fneur.2025.1612955 (PMC12527863; doi:10.3389/fneur.2025.1612955)
Supplement: Supplementary file 1 [file Data_Sheet_1.pdf]

Supplementary Material

Supplementary 1. Ecological Momentary Assessment - Therapist Version (EMA-T).

VALUTAZIONE SETTIMANALE MUSICOTERAPIA ORCHESTRALE

(Da compilare il giorno successivo alla sessione orchestrale)

Data: ___/___/____ Codice Paziente: _______ Settimana n°: T___

Per ogni item, indicare quanto il comportamento descritto è stato osservato nella sessione orchestrale della settimana appena trascorsa, tramite la seguente scala:

1=Mai | 2=Raramente | 3=Qualche volta | 4=Spesso | 5=Sempre

A. ABILITA' COGNITIVE

1. Il paziente rimane concentrato per tutta la durata della sessione?	
2. Il paziente accoglie nuove sfide musicali proposte dal direttore?	
3. Il paziente si adatta a modifiche nel ritmo e nella dinamica musicale in tempo reale?	
4. Il paziente rimane ancorato, nonostante abbia ricevuto nuove istruzioni, ai precedenti compiti musicali?	
5. Il paziente si prepara adeguatamente alla sessione? Es: *raggiungendo la propria postazione, preparando lo strumento, gli spartiti e i materiali di lavoro che serviranno.*	
6. Il paziente riesce a focalizzarsi sui compiti musicali e a non farsi distrarre da altri input esterni?	
7. Il paziente riesce simultaneamente a leggere lo spartito e ad eseguire la partitura?	
8. Il paziente riesce simultaneamente a suonare lo strumento e a seguire lo sguardo e i segnali del direttore?	
9. Il paziente utilizza strategie di autocorrezione in tempo reale?	
10. Il paziente si mostra consapevole dei propri errori?	
11. Il paziente possiede prontezza nella ricezione e nella risposta a nuovi stimoli?	
12. Il paziente risponde in modo discrepante alle intenzioni musicali altrui?	
13. Il paziente suona durante spazi vuoti in modo inappropriato senza rispettare le pause della partitura?	
14. Il paziente ricorda le nuove istruzioni impartite nel breve termine durante l'esecuzione?	
15. Il paziente ricorda alcuni passaggi delle partiture eseguite a distanza di pochi minuti?	
16. Il paziente ricorda alcuni passaggi delle partiture eseguite anche distanza di giorni?	
17. Il paziente si dimostra orientato nello spazio orchestrale? *Es: riconosce il proprio posto e quello degli altri?*	

B. ABILITA' SOCIALI E COMUNICATIVE

1. Il paziente interagisce spontaneamente con gli altri membri dell'ensemble?	
2. Il paziente esegue il proprio ruolo senza sovrapporsi agli altri, rispettando la gerarchia orchestrale?	
3. Il paziente segue gli sguardi e i gesti del direttore?	
4. Il paziente risponde adeguatamente ai segnali non verbali degli altri strumentisti?	
5. Il paziente durante le interazioni sociali con l'ensemble mostra segni di evitamento?	

6. Il paziente riesce a seguire e a mettere in pratica i segnali gestuali del direttore senza ulteriori indicazioni verbali?	
7. Il paziente riconosce e aiuta i suoi compagni nei momenti di difficoltà?	
8. Il paziente accetta cambi di ruolo all'interno dell'orchestra?	
9. Il paziente mostra sorriso sociale in risposta a momenti positivi durante la sessione?	
10. Il paziente mantiene una postura orientata verso il gruppo?	

C. REGOLAZIONE EMOTIVA E MOTIVAZIONE

1. Il paziente si inserisce spontaneamente nelle attività senza necessità di sollecitazione?	
2. Il paziente inizia un'adeguata azione musicale senza istruzioni dirette?	
3. Il paziente mostra una mimica facciale coerente con il contesto musicale?<br>*Es: musica allegra= sorriso, etc.*	
4. Il paziente si mostra interessato verso le attività svolte per tutto l'arco della sessione?	
5. Il paziente ha reazioni eccessive durante la sessione?<br>*Es: pianto inconsolabile, scoppi d'ira, riso incontrollabile, etc.*	
6. Il paziente manifesta episodi di freezing (blocco) durante le sessioni?	
7. Il paziente manifesta rabbia o tristezza a seguito di errori esecutivi?	
8. Il paziente manifesta ansia quando vengono proposte nuove sfide musicali?<br>*Es: accelerazione del respiro, tensione muscolare, sudorazione, vomito, etc.*	

D. ABILITA' MOTORIE

1. Il paziente mostra precisione nei movimenti delle dita e delle mani nel contesto orchestrale?	
2. Il paziente ha una presa adeguata sullo strumento?	
3. Il paziente mostra rigidità o difficoltà nei passaggi tecnici?	
4. Durante la seduta il paziente assume posture scorrette che inficiano l'esecuzione?	
5. Durante la seduta il paziente riesce a mantenere una buona postura per l'intera sessione?	
6. Il paziente riesce a mantenere un buon equilibrio tra controllo e rilassamento nelle esecuzioni tecniche?	
7. Il paziente coordina adeguatamente braccia e gambe durante l'esecuzione?	
8. Il paziente esegue movimenti ripetitivi e non funzionali al contesto?	
9. Il paziente ha difficoltà a regolare il respiro in funzione dell'esecuzione?	

E. SINCRONIZZAZIONE

1. Il paziente riesce a mantenere un movimento ritmico costante?	
2. Il paziente modula il volume in base all'insieme orchestrale?	
3. Il paziente si adatta alla dinamica musicale del contesto?	
4. Il paziente modula il tempo e l'intensità in base all'ensemble?	
5. Il paziente mostra resistenza nel sincronizzarsi con i cambiamenti dell'ensemble?	
6. Il paziente riesce a correggere la propria esecuzione in tempo reale?	
7. Il paziente si mantiene allineato con la sezione strumentale di riferimento?	

8. Il paziente commette errori di entrata o di uscita?<br>*Es: ritardi o uscite premature.*	
9. Il paziente necessita di segnalazioni esterne per riuscire a sincronizzarsi con l'ensemble?	

Note sulle osservazioni settimanali:

ELEMENTI SIGNIFICATIVI DA RIPORTARE

Supplementary 1. The Ecological Momentary Assessment – Therapist Version (EMA-T) is a structured observational tool developed by clinical experts to monitor motor, cognitive, socio-communicative, emotional-regulatory, motivational, and synchronization domains during I-SOUND interventions. EMA-T is completed by an independent and blinded clinical assessor (clinical psychologist) within 24 h post-session, with quality assurance based on co-rating (20–25% of cases) and inter-rater reliability (ICC ≥0.80). Administered weekly over 20 weeks, EMA-T supports trajectory mapping and process modeling under ecologically valid conditions. Adapted from ecological momentary assessment frameworks [53].
