# Supplementary material for: Inclusive orchestral music therapy according to the Euterpe Method: a multimodal framework for neurodevelopmental disorders
Source: Front Neurol. 2025 Oct 2;16:1612955. doi: 10.3389/fneur.2025.1612955 (PMC12527863; doi:10.3389/fneur.2025.1612955)
Supplement: Supplementary file 2 [file Data_Sheet_2.pdf]

Supplementary Material

Supplementary 2. Ecological Momentary Assessment - Parent Version (EMA-P).

DIARIO SETTIMANALE GENITORI

MUSICOTERAPIA ORCHESTRALE (OMT EM)

(Da compilare il giorno successivo alla sessione orchestrale)

Data: ___/___/____ Codice Paziente: _______ Settimana n°: T___

Non esistono risposte giuste o sbagliate. Vi invitiamo a leggere ogni domanda e a indicare quanto rispecchia le osservazioni fatte tra l'ultima sessione di musicoterapia orchestrale e quella appena terminata. Scegliete il numero che meglio descrive la situazione tramite questa scala:

1=Mai | 2=Raramente | 3=Qualche volta | 4=Spesso | 5=Sempre

A. ABILITÁ COGNITIVE

1. Mantiene la concentrazione su attività quotidiane fino alla fine?	
2. Accetta cambiamenti nella routine quotidiana?<br>*Es: andare a dormire ad un'ora diversa, cambiare posto a tavola, etc.*	
3. Rispetta turni e attende il suo momento in attività quotidiane?	
4. Si prepara alle attività che deve svolgere?<br>*Es: sa che potrà guardare la tv dopo cena; quindi, si impegna a fare il resto delle attività prima di cena.*	
5. Mantiene la concentrazione su determinati stimoli (*es: tv, gioco, conversazione, musica*) senza farsi distrarre da altri input esterni?	
6. Riesce a fare più attività contemporaneamente?<br>*Es: seguire una conversazione o guardare la tv e contemporaneamente mangiare.*	
7. Trova soluzioni alternative quando si presenta una difficoltà?	
8. Si mostra consapevole dei propri errori dopo aver svolto un'attività quotidiana?<br>*Es: aver fatto cadere un oggetto a terra.*	
9. Mostra di possedere prontezza a ricevere e rispondere a stimoli esterni?<br>*Es: si gira se chiamato o se toccato sulla spalla.*	
10. Esegue azioni di precedenti attività che non sono più funzionali al contesto?<br>*Es: dopo aver ascoltato musica e battuto le mani, continua a batterle anche dopo che l'ascolto della musica è terminato e bisogna fare altro.*	
11. Risponde in maniera adeguata alle intenzioni delle persone?<br>*Es: guardando un film mostra di capire le intenzioni di un personaggio cattivo tramite espressioni e verbalizzazioni di paura.*	
12. Se gli viene impartita un'istruzione, tiene a mente i passaggi che deve fare fino al completamento dell'attività?<br>*Es: esempio di istruzione "Poggia la forchetta accanto al piatto, bevi un po' d'acqua e pulisci la bocca con il fazzoletto".*	
13. Ricorda istruzioni anche a distanza di ore/giorni?	
14. Ricorda istruzioni anche a distanza di pochi minuti (*Es:10 min*)?	
15. Si mostra orientato negli spazi di casa e negli spazi a lui familiari?	

Note sulle osservazioni settimanali:

B. ABILITA' SOCIALI E COMUNICATIVE

1. Interagisce spontaneamente con familiari e amici?	
2. Rispetta i turni nelle conversazioni o nelle attività?	
3. Segue lo sguardo e i gesti dei familiari o degli amici?<br>*Es: guarda negli occhi durante una conversazione o segue la direzione di una mano che indica qualcosa.*	
4. Risponde adeguatamente ai segnali non verbali?<br>*Es: fa silenzio se qualcuno si preme un dito sulla bocca, si avvicina se qualcuno fa segno di avvicinare.*	
5. Mostra segnali di evitamento durante interazioni sociali, come una festicciola in casa, la visita di persone esterne alla famiglia o attività all'aperto in presenza di altra gente?<br>*Es: sta lontano dalle persone o vuole tornare a casa.*	
6. Riconosce se un familiare si trova in difficoltà e lo aiuta o sostiene?<br>*Es: se qualcuno si fa male cerca di aiutare o da un abbraccio ad una persona che piange.*	
7. Accetta cambi di ruolo nelle situazioni quotidiane?	
8. Sorride in risposta a momenti positivi insieme agli altri?<br>*Es: riceve un complimento da una persona, capita qualcosa di divertente a qualcuno a lui vicino, etc.*	
9. Mantiene una postura orientata verso il gruppo, ad esempio in una festicciola?	

Note sulle osservazioni settimanali:

C. REGOLAZIONE EMOTIVA E MOTIVAZIONE

1. Compie spontaneamente attività quotidiane senza necessità di sollecitazione o istruzioni?	
2. Mostra una mimica facciale coerente con il contesto?<br>*Es: sorride o si intristisce quando guarda se il contesto è allegro o triste.*	
3. Mantiene l'interesse verso le sue attività quotidiane fino al loro completo svolgimento?	
4. Ha reazioni eccessive durante le giornate?<br>*Es: pianto inconsolabile, scoppi d'ira, riso incontrollabile, etc.*	
5. Manifesta episodi di blocco durante alcune attività?<br>*Es: si ferma improvvisamente e non si muove, né parla per svariati minuti.*	
6. Manifesta rabbia o tristezza a seguito di errori in attività quotidiane?<br>*Es: versa accidentalmente dell'acqua sul tavolo o fa cadere del cibo fuori dal piatto.*	
7. Manifesta ansia quando vengono proposte nuove attività che modificano la routine quotidiana?<br>*Es: respira velocemente, diventa teso, etc.*	

Note sulle osservazioni settimanali:

D. ABILITA' MOTORIE

1. Mostra precisione nei movimenti delle dita e delle mani?<br>*Es: con le posate, i vestiti e gli oggetti in generale.*	
2. Mostra di avere una presa adeguata sugli oggetti?<br>*Es: li tiene bene senza farli cadere o traballare.*	
3. Mostra rigidità durante movimenti mano-braccio?<br>*Es: afferrare un oggetto, manipolarlo e poi riporlo nuovamente.*	
4. Assume durante la giornata posture scorrette che inficiano le attività quotidiane?<br>*Es: non sta con la schiena e le spalle dritte a tavola e questo rende difficoltosa la nutrizione.*	
5. Mantiene un buon equilibrio nelle attività quotidiane?<br>*Es: camminare, stare seduti senza cadere lateralmente, tenere un bicchiere senza rovesciare il contenuto.*	
6. Esegue movimenti ripetitivi e non funzionali al contesto?<br>*Es: batte ripetutamente la mano sul tavolo, si dondola, etc.*	

Note sulle osservazioni settimanali:

E. SINCRONIZZAZIONE

1. Mantiene il ritmo quando ascolta la musica o partecipa a giochi musicali?<br>*Es: batte le mani, oscilla la testa ritmicamente*	
2. Modula il volume della voce a seconda dei contesti?<br>*Es: a casa parla/vocalizza liberamente, in giro parla/vocalizza con un volume moderato*	
3. Riesce a calibrare i movimenti in base al contesto?<br>*Es: cammina velocemente quando è fuori, rallenta quando entra in un negozio/sala d'aspetto etc.; si guarda intorno velocemente se è circondato da tante persone, si guarda intorno lentamente se circondato da poche persone.*	

Note sulle osservazioni settimanali:

ELEMENTI SIGNIFICATIVI DA RIPORTARE

Supplementary 2. The Ecological Momentary Assessment – Parent Version (EMA-P) is a complementary caregiver-report tool designed to capture changes in motor, cognitive, socio-communicative, emotional-regulatory, motivational, and synchronization domains in everyday contexts. EMA-P is completed by the caregiver/parent within 24–48 h after each intervention session, across 20 consecutive weeks, to provide ecologically grounded longitudinal data. Together with EMA-T, EMA-P contributes to delineating therapeutic trajectories and correspondence maps, enhancing external validity and real-world applicability. Both EMA-T and EMA-P are conceptually adapted from ecological momentary assessment frameworks [53]. In addition, weekly EMA-P entries include structured observational notes on home-based practice, covering instrument assembly, exercise review, cleaning, and maintenance, thereby documenting ecological continuity beyond formal IMT and OMT sessions.
