# Supplementary material for: Inclusive orchestral music therapy according to the Euterpe Method: a multimodal framework for neurodevelopmental disorders
Source: Front Neurol. 2025 Oct 2;16:1612955. doi: 10.3389/fneur.2025.1612955 (PMC12527863; doi:10.3389/fneur.2025.1612955)
Supplement: Supplementary file 3 [file Data_Sheet_3.pdf]

Supplementary Material

Supplementary Table 1. TIDieR checklist for the I-SOUND intervention.

TIDieR item	Description (I-SOUND)	Relevance for outcomes
**1. Brief name**	Inclusive Orchestral Music Therapy – I-SOUND (IMT, OMT, and MIT-P).	Clear identification of the intervention and its modular components ensures reproducibility and cross-study comparability.
**2. Why (Rationale)**	The intervention is aimed at fostering adaptive resilience and stratified functional targeting in children and adolescents with NDD. It specifically addresses motor planning, executive control, socio-emotional regulation, and predictive timing mechanisms, based on principles of adaptive plasticity and multimodal enrichment.	Provides the neurofunctional rationale linking intervention modules with targeted clinical outcomes, aligning with frameworks of experience-dependent plasticity.
**3. What (Materials)**	Standardized orchestral instruments adapted for accessibility, multimodal cue cards and visual prompts, calibrated sound level meter for continuous monitoring (aligned with WHO/NIOSH safe listening standards), and recording devices for fidelity checks.	Ensures uniformity of materials across sites; acoustic safety and multimodal cues are critical for ecological validity and patient tolerance.
**4. What (Procedures)**	• IMT sessions are clinically adapted from the EM Active algorithm, focusing on individualized sensorimotor and cognitive training.<br>• OMT sessions employ clinically adapted orchestral conducting techniques to structure group performance, role differentiation, and hierarchical synchronization.<br>• The MIT-P module regulates transfer across contexts.<br>• Pre-specified decision rules govern role rotation, progression of tempo/dynamics (±5–10% based on EMA results), and adaptation of tasks to clinical status.	Describes the operative sequence, ensuring replicability and fidelity; explicit decision rules link intervention dynamics with measurable outcomes.
**5. Who provides**	Licensed music therapists with formal orchestral conducting training, supported by certified health professionals (neurologists, neuropsychologists, physiatrists, occupational therapists). All providers underwent >60 hours of standardized	Provider expertise and standardized training ensure treatment fidelity and clinical safety.

	training, including supervised practice and fidelity calibration.	
**6. How**	Delivered face-to-face in person. IMT precedes OMT within 1–5 days, with MIT-P acting as a transversal regulatory module. Caregivers (primarily parents) assist participants on non-therapy days with instrument-related tasks (assembly, cleaning, maintenance) and reinforce therapist- and conductor-assigned instructions at home. These activities promote ecological continuity, treatment adherence, and sustained engagement beyond formal sessions. Video-based supervision and structured fidelity checklists are used to monitor adherence.	Strengthens internal/external validity and multicenter replicability.
**7. Where**	Sessions take place in hospital-based rehabilitation units, conservatory auditoria, and community-based extra-hospital centers. All spaces are accessible and acoustically treated to meet safety standards (RT60 < 1.0 s; LAeq ≤ 85 dB).	Describes standardized environmental requirements for safety, accessibility, and ecological validity across sites.
**8. When and how much**	• Phase 1 (observational, ongoing): standard 20-week cycle with weekly IMT-OMT macro-sequence, separated by a 1–5-day interval. IMT: ~20 × 60 min; OMT: ~20 × 90 min. Monitoring: weekly EMA-T/EMA-P ×20. Total cumulative dose: ~3,000 min (IMT 1,200 + OMT 1,800).<br>• Phase 2 (planned RCT): same structure and dosage; standardized assessments at T0 (baseline), T1 (10 weeks), T2 (20 weeks), T3 (12 months), and T4 (24 months). EMA will not be included in efficacy analyses.	Provides total intervention dose and timing, critical for dose-response interpretation and outcome analysis.
**9. Tailoring**	Instrument allocation and role assignments are tailored to clinical profile, sensory tolerance, and targeted functional domains. Decision rules specify adaptations (e.g., substitution of instrument if sensory intolerance >30% EMA reporting; rotation of orchestral role after ≥2 consecutive successful trials).	Clarifies personalization rules, ensuring transparency and reproducibility of tailoring while linking directly to outcome measures.
**10. Modifications**	Pre-specified adjustments guided by performance data and EMA feedback (e.g., modification of task complexity, pacing, or orchestral role). Actual modifications	Maintains transparency between planned and actual modifications, supporting internal validity.

	emerging during the study are documented separately in case report forms.	
**11. How well (planned)**	Fidelity plan included >60 hours of provider training, supervised practice, and calibration. Standardized fidelity checklist covers session structure, adherence to algorithmic steps, orchestral role distribution, and caregiver engagement.	Ensures pre-trial standardization and provides objective domains for fidelity monitoring.
**12. How well (actual)**	Fidelity was assessed weekly by two independent raters. Inter-rater reliability was calculated using ICC(2,k) with 95% confidence intervals. A priori threshold: ICC ≥0.80 (good); ≥0.90 (excellent). Protocol deviations triggered immediate retraining and re-calibration.	Provides transparency and quantifiable thresholds for fidelity, strengthening internal validity and reproducibility.
Abbreviations: EM Active = Euterpe Method Active algorithm; EMA-T = Ecological Momentary Assessment – Therapist-reported; EMA-P = Ecological Momentary Assessment – Parent-reported; ICC = Intraclass Correlation Coefficient; I-SOUND = IncluSive Orchestral mUsic therapy accordiNg to the euterpe method; IMT = Individual Music Therapy; LAeq = Equivalent continuous sound level; MIT-P = Multidirectional and Iterative Transfer Process; NDD = Neurodevelopmental Disorders; NIOSH = National Institute for Occupational Safety and Health; OMT = Orchestral Music Therapy; RCT = Randomized Controlled Trial; RT60 = Reverberation time (sound decay by 60 dB); WHO = World Health Organization.		

Supplementary Table 1. Adapted from the TIDieR checklist [20]. The table summarizes the I-SOUND modules (IMT, OMT, MIT-P), their clinically adapted procedures, and expected neurofunctional outcomes, to support methodological transparency and replicability.
